# The Relationship between Coping and Expressed Emotion in Substance Users

**DOI:** 10.3390/jcm11195766

**Published:** 2022-09-29

**Authors:** Grace Y. Wang

**Affiliations:** School of Psychology and Wellbeing, University of Southern Queensland, Darling Heights, QLD 4350, Australia; grace.wang@usq.edu.au

**Keywords:** expressed emotion, coping, substance use, stress, criticism

## Abstract

The involvement of family is an integral part of the recovery process, and the use of adaptive coping strategies has an important implication for treatment outcomes. Little research to date has examined the relationship between coping and family dynamics in substance users, although this may help to unravel the mechanism underlining the increased risk of relapse for individuals from critical family environment. The aim of the present research was to assess the association between the level of expressed emotion (LEE) (i.e., criticism), coping style, and psychological distress (i.e., anxiety, depression) in people with substance use disorder (SUD). Compared to control subjects, persons with SUD reported less use of rational coping and detached coping, and perceived greater criticism and irritability from family. A higher degree of family criticism and lack of emotional support was associated with greater use of emotional and avoidance coping in persons with SUD, while psychological distress was more related to rational and detached coping. The present study reveals the unique connection between family relationships, coping and psychological distress, implicating the need to address the influence of family relationships and stress on persons’ coping in SUD treatment.

## 1. Introduction

The level of expressed emotion (LEE) (e.g., criticism) within a household is important in the treatment of individuals with substance use disorder (SUD) as the involvement of family is an integral part of the recovery process [1]. Specifically, research shows high LEE within relatives and the household being a predictor for lesser changes and a worse off treatment outcome as opposed to individuals with low scoring expressed emotion (EE) relatives and households [2,3,4]. EE encompasses hostility, criticalness and emotional over-involvement, all of which can hinder the effectiveness of the treatment in preventing relapse [1]. Thus, EE is important to consider when implementing treatment for individuals with SUD.

Our recent research shows altered sensitivity to criticism in persons with SUD. Compared to healthy control subjects, individuals with a history of SUD tend to rate criticism as less arousing and those with SUD co-occurring with mental disorders rate criticism as more self-relevant [5]. The rating of criticism is positively correlated with schizotypy traits and depression, and the age of onset of substance use was a significant predictor of the arousal of criticism [5]. Our findings implicate the complexities of perceived EE in substance users and the link between EE and relapse might not always be unidirectional as claimed in previous literature, e.g., [4] and there are possible dynamic changes in perceived EE associated with individual characteristics. 

Coping is a central process in adaptation and survival and refers to the basic process of how individuals detect, learn and deal with stressful events [6]. It plays an important influence in the development, course, and treatment outcome of SUD [7,8]. A variety of coping strategies are used by individuals depending on the stressful event and the perceived threat to the environment. The approach and strategy that an individual adopts when dealing with a stressor or stressful environment can amplify the effects of stress on the resilience and psychopathology of individuals [6,9]. There is a multitude of coping strategies and distinctions in the way individuals cope with stressful events, stimuli, or emotions. The first distinction in coping is between problem-focused and emotion-focused coping. Problem-focused coping refers to the strategy in which coping is targeted toward a stressor and involves an individual taking measures to avoid the stressor or decrease the impact of the stressor [10]. Emotion-focused coping is the process in which an individual focuses effort on reducing the emotional impact of stressful events such as fear, stress, and anxiety [11]. A further distinction in coping styles is between engagement and disengagement coping. Engagement coping refers to the process in which an individual will actively confront and deal with the emotions arising from a stressful event [10]. While disengagement coping refers to strategies that are used to divert attention away from the stressor and any resulting negative emotions [12]. 

The use of engagement coping strategies is associated with lower substance use and better treatment adherence [7], while reliance on cognitive avoidance coping could comprise the positive association between self-efficacy and treatment outcome [8]. Preference for use of disengagement strategies has been found in patients with SUD and those with dual diagnosis (SUD and mental disorder) [13]. In particular, persons with SUD and major depressive disorder show greater use for disengagement strategies such as social withdrawal and lower in engagement approaches such as problem solving, and social support when facing stressful events [14]. It has been argued that there is a need to tailor treatment programs to reduce such disengagement strategies and to train patients to take active problem-solving approaches in coping [13]. 

However, the manner in which individuals respond to stressful events can be affected by the EE attitudes of family members. Evidence suggests that persons’ appraisal and engagement with negative affective behaviours from family members influence their preferred coping strategies [15]. Persons from high-EE homes tend to use emotion-based confrontational coping methods, such as expression of anger and frustration, compared to those from low-EE home, and the preference of this coping style can be found during both family conflicts and non-familiar, societal stressors [15]. In contrast, individuals from low-EE family are likely to use avoidance and denial to minimise the impact of stress in their lives [15]. 

Little research to date examines the relationship between coping and EE in substance users, although this may help to unravel the mechanism underlining the increased risk of relapse for individuals from high-EE households. Research with persons with alcohol dependence shows no correlation between coping behaviours and level of expressed emotions, but the age of the first intake of alcohol is an important predictor of coping and perceived EE [16]. Nevertheless, it should be noted that coping behaviours assessed in this research are solely related to alcohol drinking which may not accurately reflect individuals’ usage tendencies in dealing with stressful situations, e.g., family conflicts. The aim of the present research was to determine the impact of the history of substance use on LEE and coping style and assess the association between the LEE, coping style, individuals’ history of substance use. More specifically, it was hypothesised that:

1. An individual with SUD would report a greater preference for using emotional and avoidance coping styles compared to non-drug using controls;
2. Emotional and avoidance coping would be positively correlated with the LEE and depression in substance users;
3. Coping styles would be predicted by age of onset of substance use and length of drug abstinence.

## 2. Methods

### 2.1. Participants and Procedure 

Adults with a history of SUD (*n* = 42) were recruited via advertisement in a drug rehabilitation centre and snowball sampling. Both participants and staff at the drug rehabilitation centre were encouraged to promote this study with their network. Substance users were included if they met the following criteria: (1) aged ≥ 18 years; (2) have been diagnosed SUD; and (3) able to give informed consent. Non-using adults (*n* = 56) were recruited as controls through advertisements posted on a community notice board and social media. Control subjects must meet the same inclusion criteria as subjects with SUD, except for drug use. Effort was made to match the gender and age between groups during recruitment.

The study protocol was approved by the institutional Ethics Committee (Auckland University of Technology Ethics Committee, 19/81, dated 14 May 2019). Signed consent was obtained from all participants prior to the study commencing. The eligibility of participants was checked prior to participating in the study. Options for paper based and online surveys were offered. The study data for the SUD group was primarily gathered using a paper-pencil survey named “Perceived expressed emotion in people with substance use disorder” (Supplementary Material One) and assistance was given when participants experienced difficulty understanding question items, which effectively enhanced the response rate and participants’ accuracy in question interpretation.

### 2.2. Measures

#### 2.2.1. Level of Expressed Emotion (LEE) 

The LEE, a 38-item 4-point Likert scale, assesses the perceived emotional climate within an individual’s most influential personal relationships [17]. The scale appraised perceived criticism, intrusiveness, lack of emotional support, and irritability [18,19]. The internal reliability for subscale Criticism, Irritability, Intrusiveness, and Lack of Emotional Support ranged from good to excellent, with the Cronbach’s alphas being 0.75, 0.80, 0.82, and 0.93, respectively, [20]. The Cronbach’s alphas for the subscales in the present study were 0.64 for Criticism, 0.74 for Irritability, 0.83 for Intrusiveness, and 0.89 for Lack of Emotional Support.

#### 2.2.2. Coping Styles Questionnaire (CSQ)

This 60-item scale measures two adaptive coping styles (detached and rational) and two maladaptive coping styles (emotional and avoidance) [21]. Good internal consistency and reliability of the CSQ has been reported, with coefficient alpha 0.85, 0.90, 0.74, and 0.69 for rational coping, detached coping, emotional coping, and avoidance coping, respectively, [21]. The Cronbach’s alphas for the subscales in the present study were 0.92 for rational coping, 0.84 for detached coping, 0.90 for emotional coping, and 0.78 for avoidance coping.

#### 2.2.3. Psychological Distress: Depression, Anxiety and Stress Scale (DASS-21)

It assesses three dimensions of mental wellbeing (seven items each) over the past week: depression, anxiety, and stress [22]. Good convergent and discriminant validity, and reliability of DASS-21 and its subscales have been reported with a Cronbach Alpha of 0.72 for Depression, 0.77 for Anxiety, 0.70 for Stress subscale and 0.88 for the overall scale [23]. The Cronbach’s alpha for the subscales in the present study were 0.80 for Anxiety, 0.90 for Depression, and 0.88 for Stress.

### 2.3. Data Analysis

A priori power analyses were conducted using G*Power [24]. For the independent *t*-test, a sample size of 45 would have 80% power to detect a medium effect size of d = 0.60 with a two tailed α of 0.05. 

Group comparisons for LEE and coping measures were performed using an independent *t*-test. The association between LEE and coping was explored in each group, respectively, using Pearson correlations. Linear regression was performed to determine the relative effects of age of onset of substance use and days of drugs abstinence on predicting coping preference in the SUD group. Log transformation was applied to days of drug abstinence before it was inputted into the model due to its skewness. Statistical analyses were performed via IBM SPSS Statistics (version 27, IBM, Armonk, NY, USA).

## 3. Results

### 3.1. Demographic and Clinical Characteristics

The demographic and clinical characteristics of the groups are shown in Table 1. There was a significant age difference between the SUD and control groups (t = 4.57, *p* < 0.001), with older participants in the SUD group. There was no significant gender difference between the groups. 

#### Group Differences in LEE, Coping and Psychological Distress

Table 2 summarises the means and standard deviations for the LEE, coping and psychological distress in the clinical and health community samples. Comparison of the scores of individuals with SUD with those of the health samples, as estimated by means of Cohen’s d effect size estimate, shows that persons with SUD reported less use of rational coping (t = −5.2, *p* < 0.001, d = 1.06) and detached coping (t = −2.1, *p* = 0.04, d = 0.42). Furthermore, the persons with SUD perceived more criticism (t = 2.91, *p* = 0.005, d = 0.59), and greater irritability (t = 1.67, *p* = 0.05, d = 0.35), and reported greater level of depression (t = 3.5, *p* < 0.001, d = 0.72), anxiety (t = 2.6, *p* = 0.01, d = 0.54) and stress (t = 2.5, *p* = 0.01, d = 0.52). 

However, emotional coping and avoidance coping, LEE intrusiveness, and lack of emotional support were not different between the groups. 

### 3.2. Relationship between Coping Style, LEE and Psychological Distress

The correlation coefficients between the variables are presented in Table 3. Coping style was related to LEE and psychological distress, however there were similarities but differences between the SUD and non-drug using groups. The association with LEE were only found in emotional and avoidance coping styles, and there was an absence of association between adaptive coping (including rational and detached coping) and LEE in both groups. However, either emotional coping or avoidance coping was positively correlated with LEE criticism and/or intrusiveness and irritability in the health samples, while these two coping styles were positively correlated with LEE criticism and/or lack of emotional support in the SUD group. 

Furthermore, strong associations between psychological distress (including anxiety, depression, and stress) and rational and detached coping were found in the SUD group, which were absent in the non-drug using group. Greater psychological distress was associated with less use of rational coping and detached coping. Age onset of substance use was only correlated with avoidance coping, while the day of abstinence was correlated with all coping styles, apart from detached coping.

### 3.3. Prediction of Coping Style by the Age of Onset of Substance Use and Days of Substance Abstinence in the SUD Group

Linear regression analyses showed that abstinent days was the significant solo predictor for emotional coping, *F* (2, 38)  =  4.50, *p*  =  0.02, *R*^2^  =  0.19, β = −5.03, and avoidance coping, *F* (2, 38)  =  5.02, *p*  = 0.01, *R*^2^  =  0.21, β = −2.32. Whilst age of onset of substance use, and days of substance abstinence combine, the regression model did not significantly predict the rationale and detached coping (Table 4). 

## 4. Discussion

Coping style has been proposed as one of the mechanisms which might underlie early onset of substance use and clinical severity [25,26]. However, coping involves not only individual behavioural and cognitive efforts, but also an interaction between individuals and their environment [27]. The present study was to reveal the interplay between coping, family environment and psychological distress in substance users who often experience greater perceived criticism than those without SUD. The present study found that although persons with SUD reported less use of rational coping and detached coping, their use of emotional coping and avoidance coping strategies during stressful situation was not different from non-drug using controls. This finding partially supports the previous research [14], suggesting that persons with SUD exhibit deficit coping strategies during treatment. However, inconsistent with the previous research [13] which has shown a greater preference for maladaptive coping, such as emotional coping and avoidance coping styles, in people with SUD relative to those without a history of SUD, the present finding suggests that when persons with SUD undertaking SUD treatment, their preferences for maladaptive coping styles are not different from those without SUD. The reasons for this could be related to the positive influence associated with current treatment involvement in our SUD group, who receive consistent professional support and counselling help, or improved somatic and psychological status in patients following drug abstinence. For example, emotional regulation and specific coping strategies, e.g., problem solving, have been frequently included in daily group therapy sessions.

As expected, a higher degree of perceived criticism and lack of emotional support from family were associated with greater use of emotional and avoidance coping in persons with SUD in the present study. Furthermore, anxiety, depression and stress are also associated with emotional and avoidance coping. Evidence suggests that social interaction particularly with the family influences the adaptation of a patient towards chronic disease [28]. In line with previous research [2,5], the present study found that the participants in the SUD group reported a greater level of perceived criticism than non-drug using controls. High levels of criticism and lack of emotional support may reflect family struggles in coping with the stress associated with patients’ substance use in a constructive manner (e.g., successfully managing stress but the result is dysfunction and unproductive), and these responses could lead patients with increased psychological distress and influence their appraisals of coping efficacy [29]. Research suggests that critical responses on persons’ coping efforts could result in persons evaluating the efforts they have made in an unfavourable light. As a result, the persons become more likely to engage in avoidance when faced with challenges [30]. In line with this, the present findings implicate that people with a greater preference for maladaptive coping styles are more likely are those from unhealthy family environments charaterised by hostile interactions and ineffective conflict management, and unfortunately, persons with SUD are more likely to view their family environment being negative and lack of empathy. 

In contrast with non-drug using controls who showed a weak association between psychological distress (including stress, anxiety, and depression) and adaptive coping (including rational and detached coping styles), a greater level of psychological distress was associated with less use of adaptive strategies. Stress can be beneficial in a minimal amount, but when it becomes unmanageable, stress could damage a person’s health and wellbeing, affecting learning, decision-making and thinking. The links between addiction and stress have been found at multiple levels, suggesting substance use is often initiated as a coping mechanism for stress [31], and enhance retrieval of drug-related memories for stress relief during abstinence [32]. Persons who lack adaptive coping skills are more likely to engage with substance use [33]. The present findings replicate this pattern of findings in a sample of SUD treatment seekers, suggesting an inverse relationship between psychological distress and adaptive coping. However, it should be noted that this may not be the case in people without a history of SUD. 

Furthermore, age onset of substance use, and length of abstinence also play a role in persons’ coping. The present findings suggest that early exposure to the substance is associated with greater use of maladaptive coping, while drug abstinence is likely to help to reduce this tendency. This might be related to the negative impact of substance intoxication and withdrawal syndrome on emotional vulnerability and cognition [34]. With a period of abstinence maintenance, some of the deficits might be reduced. Improved coping skills have also been reported in patients undertaking SUD treatment [35]. 

The present study comes with Its limitations that need to be acknowledged. The research was a cross-sectional study. Thus, no causality can be inferred. However, the present study indicates that family environment and stress could influence a person’s coping style while they are engaged in SUD treatment and their effects might be different from what we have known in the general population. Future research could look at a longitudinal study to detect changes as persons’ recovery from SUD may be fluid. Furthermore, the present study relied on self-report measures which present the opportunity to create bias as the participants may under or overestimated the intensity of engagement in certain behaviours, e.g., family criticism and coping tendency. Future work may support self-report scales with evidence derived from sources such as clinicians’ observations and experimental behavioural data from participants. Moreover, it was a limitation that there were small sizes across groups. Despite the limitations, the present study adds to the knowledge of SUD by revealing the connection between family relationship, coping and psychological distress. To improve the effectiveness of coping-focused prevention approach in SUD treatment, the influence of family relationship and stress on persons’ coping need to be highlighted. 

## 5. Conclusions

Individual coping style is closely associated with family dynamics and psychological distress in substance users. The impact of family communication and personal negative emotional status on coping tendencies needs to be highlighted in SUD treatments. 

## Figures and Tables

**Table 1:** Participant demographics and history of SUD.

Characteristics		SUD (*n* = 42)	Control (*n* = 56)
Age (years)		35.5 ± 8.2	28.8 ± 6.4
Sex		Male: 29	Male: 29
Age of onset of substance use		13.7 ± 4.1	N/A
Choice of substance			
	Methamphetamine	19	
	Alcohol	12	
	Polydrug use	11	
Years of substance use		15.1 ± 7.7	
Days of abstinence		450 ± 898	

Note: Polydrug use includes alcohol, methamphetamine, cannabis, and opiates. SUD: Substance use disoder.

**Table 2:** Means and standard deviations of outcome measures.

	SUD Mean (SD)	Control Mean (SD)
LEE Criticism	11.64 (2.91)	9.77 (3.34)
LEE Intrusiveness	16.19 (5.17)	15.82 (5.00)
LEE Irritability	15.98 (4.71)	14.43 (4.31)
LEE Lack of Emotional Support	39.79 (9.89)	36.89 (14.03)
Rational Coping	22.69 (9.07)	31.41 (7.50)
Detached Coping	18.33 (7.32)	21.39 (7.19)
Emotional Coping	20.02 (9.43)	17.37 (8.73)
Avoidance Coping	18.38 (5.39)	17.65 (6.94)
Anxiety	5.52 (5.08)	3.46 (2.54)
Depression	6.79 (5.89)	3.43 (3.42)
Stress	7.86 (5.51)	5.54 (3.55)

Note: LEE: Level of Expressed Emotion; SUD: Substance use disorder; SD: Standard deviation.

**Table 3:** Relationship of own coping style with LEE scales and negative emotional states.

	SUD Group				Non-Using Control			
	Rational Coping	Detached Coping	Emotional Coping	Avoidance Coping	Rational Coping	Detached Coping	Emotional Coping	Avoidance Coping
Level of expressed emotion								
Criticism	−0.30	−0.30	0.39 *	0.001	−0.06	−0.08	0.34 *	0.12
Intrusiveness	−0.22	−0.23	0.17	0.07	0.15	0.17	0.35 **	0.39 **
Irritability	−0.18	−0.12	0.27	0.20	0.01	0.001	0.39 **	0.33 *
Lack of support	−0.25	−0.10	0.43 **	0.40 **	−0.05	−0.12	0.18	−0.05
Psychological distress								
Anxiety	−0.32 *	−0.25	0.66 **	0.58 **	−0.10	−0.03	0.52 **	0.28 *
Depression	−0.41 **	−0.32 *	0.80 **	0.59 **	−0.26	−0.16	0.72 **	0.37 **
Stress	−0.37 *	−0.36 *	0.72 **	0.63 **	−0.37 **	−0.25	0.59 **	0.29 *
Onset of age of substance use	−0.18	−0.05	−0.15	0.30 *				
Days of Abstinence	0.36 *	0.17	−0.44 **	−0.42 **				

**. Correlation is significant at the 0.01 level (2-tailed); *. Correlation is significant at the 0.05 level (2-tailed). Note: LEE: Level of Expressed Emotion; SUD: Substance use disorder

**Table 4:** Regression coefficient of substance use predictors of coping.

Variables	Unstandardized Coefficients			Standardized Coefficients	*p*
	B	SE (B)	95% CI	β	
Age of onset of susbtance use	0.24	0.35	[−0.69, 0.74]	0.01	0.95
Days of abstinence	−5.03	1.79	[−8.65, −1.40]	−0.43	0.008
Dependent Variable: Emotional coping					
Age of onset of susbtance use	0.25	0.20	[−0.15, 0.65]	0.20	0.21
Days of abstinence	−2.32	1.00	[−4.34, −0.30]	−0.36	0.03
Dependent Variable: Avoidance coping					
Age of onset of susbtance use	−0.02	0.29	[−0.60, 0.55]	−0.01	0.93
Days of abstinence	1.45	1.44	[−1.47, 4.37]	0.17	0.32
Dependent Variable: Detached coping					
Age of onset of susbtance use	−0.17	0.35	[−0.88, 0.54]	−0.08	0.63
Days of abstinence	3.75	1.78	[−0.15, 7.35]	0.34	0.04
Dependent Variable: Rational coping					

## Data Availability

Data available on request from the author.

## Supplementary Materials

The following supporting information can be downloaded at: https://www.mdpi.com/article/10.3390/jcm11195766/s1. Supplementary Material One: A copy of paper-based survey used in the present study.
