# Supplementary material for: The Relationship between Coping and Expressed Emotion in Substance Users
Source: J Clin Med. 2022 Sep 29;11(19):5766. doi: 10.3390/jcm11195766 (PMC9570536; doi:10.3390/jcm11195766)
Supplement: Supplementary file 1 [file jcm-11-05766-s001.zip › jcm-1941916-supplementary.pdf]

Supplementary material one:

Survey: Perceived expressed emotion in people with substance use disorder

Unique ID: ____________________ Age: ____________Sex___________ Onset of Substance Use (age)____

Drug/alcohol use

	Past 30 days Days	Lifetime Yrs	Use Rt of adm. ®
D1. Alcohol-any use at all			
D2. Alcohol –to intoxication			
D3. Heroin			
D4. Methadone			
D5. Other opiates/analgesics			
D6. Barbiturates			
D7. Other sedative/hyp/tranq			
D8. Cocaine			
D9. Amphetamines			
D10. Ecstasy			
D11. Cannabis			
D12. Hallucinogens			
D13. Inhalants			
D14. Nicotine			
D16. More than on substance per day (incl. alcohol)			

Route of Administration: ®1= Oral, 2=Nasal, 3=smoking, 4=Non IV inj, 5= IV inj

D17. Which substance is the major problem?

D18. How long was your last period of voluntary abstinence from this major substance? (00-never abstinent)__________mos

D19. How many months ago did this abstinence end? (00-still abstinent)____

The following are a number of statements that describe the ways in which someone may act towards you. Please indicate whether the most influential person in your life has acted in these ways during the past 3 months.

	Untrue	More or less untrue	More or less true	True
1. Calms me down when I'm upset.				
2. Is tolerant with me, even when I'm not meeting his/her expectations.				
3. Is sympathetic towards me when I'm ill or upset.				
4. Can see my point of view.				
5. Is always interfering.				
6. Can't think straight when things go wrong.				
7. Will help me when I'm upset.				
8. Makes me feel valuable as a person.				
9. Knows how to handle my feelings when I'm unwell.				
10. Understands my limitations.				
11. Often checks up on me to see what I'm doing.				
12. Is able to be in control in stressful situations.				
13. Tries to make me feel better when I'm ill.				
14. Is always nosing into my business.				
15. Hears me out.				
16. Has to know everything about me.				
17. Makes me feel relaxed when he/she is around.				
18. Accuses me of exaggerating when I say I'm unwell.				
19. Will take it easy with me, even if things aren't going right.				
20. Insists on knowing where I'm going.				

21. Is a considerate person when I'm ill.				
22. Butts into my private matters.				
23. Can cope well with stress.				
24. Is willing to gain more information to understand my condition, when I'm not feeling well.				

	Untrue	More or less untrue	More or less true	True
25. Is understanding if I make a mistake.				
26. Doesn't pry into my life.				
27. Makes matters worse when things aren't going well.				
28. Often accuses me of making things up when I'm not feeling well.				
29. Flies off the handle when I don't do something well.				
30. Gets upset when I don't check in with him/her.				
31. Gets irritated when things don't go right.				
32. Tries to reassure me when I'm not feeling well.				
33. Expects the same level of effort from me, even if I don't feel well.				
34. Is critical of me.				
35. Tries to change me.				
36. Gets annoyed when I want something from him/her.				
37. Usually agrees with me.				
38. Shows me he/she loves me.				

Stress and anxiety *

Please read each statement and place a tick "✓" to indicate how much the statement applied to you over the past week. There are no right or wrong answers. Do not spend too much time on any statement.

	Did not apply to me at all	Applied to me to some degree, or some of the time	Applied to me to a considerable degree, or a good part of time	Applied to me very much, or most of the time
I found it hard to wind down.				
I was aware of dryness of my mouth.				
I couldn't seem to experience any positive feeling at all.				
I experienced breathing difficulty (e.g., excessively rapid breathing, breathlessness in the absence of physical exertion).				
I found it difficult to work up the initiative to do things.				
I tended to over-react to situations.				
I experienced trembling (e.g., in the hands).				
I felt that I was using a lot of nervous energy.				
I was worried about situations in which I might panic and make a fool of myself.				
I felt that I had nothing to look forward to.				
I found myself getting agitated.				
I found it difficult to relax.				
I felt down-hearted and blue.				
I was intolerant of anything that kept me from getting on with what I was doing.				
I felt I was close to panic.				

**I was unable to become enthusiastic about anything.**				
**I felt I wasn't worth much as a person.**				
**I felt that I was rather touchy.**				
**I was aware of the action of my heart in the absence of physical exertion (e.g., sense of heart rate increase, heart missing a beat).**				
**I felt scared without any good reason.**				
**I felt that life was meaningless.**				

Coping Styles

Instructions: although people may react in different ways to different situations, we all tend to have a characteristic way of dealing with things which upset us. Please place a tick "✓" to indicate the way YOU typically react to stress.

	Always	Often	Sometimes	Never
1. Feel overpowered and at the mercy of the situation.				
2. Work out a plan for dealing with what has happened.				
3. See the situation for what it actually is and nothing more.				
4. See the problem as something separate from myself so I can deal with it.				
5. Become miserable or depressed.				
6. Feel that no one understands.				
7. Stop doing hobbies or interests.				
8. Do not see the problem or situation as a threat.				
9. Try to find the positive side to the situation.				
10. Become lonely or isolated.				
11. Daydream about times in the past when things were better.				
12. Take action to change things.				
13. Have presence of mind when dealing with the problem or circumstances.				
14. Avoid family or friends in general.				
15. Feel helpless-there's nothing you can do about it.				
16. Try to find out more information to help make a decision about things.				
17. Keep things to myself and not let others know how bad things are for me.				
18. Think about how someone I respect would handle the situation and try to do the same.				
19. Feel independent of the circumstances.				
20. Sit tight and hope it all goes away.				

21. Take my frustrations out on the people closest to me.				
22. 'Distance' myself so I don't have to make any decision about the situation.				
23. Resolve the issue by not becoming identified with it.				
24. Assess myself or the problem without getting emotional.				
25. Cry, or feel like crying.				
26. Try to see things from the other person's point of view.				
27. Respond neutrally to the problem.				

	Always	Often	Sometimes	Never
28. Pretend there's nothing the matter, even if people ask what's bothering me.				
29. Get things into proportion-nothing is really that important.				
30. Keep reminding myself about the good things about myself.				
31. Feel that time will sort things out.				
32. Feel completely clear-headed about the whole thing.				
33. Try to keep a sense of humour-laugh at myself or the situation.				
34. Keep thinking it over in the hope that it will go away.				
35. Believe that I can cope with most things with the minimum of fuss.				
36. Try not to let my heart rule my head.				
37. Eat more (or less) than usual.				
38. Daydream about things getting better in future.				
39. Try to find a logical way of explaining the problem.				
40. Decide it's useless to get upset and just get on with things.				
41. Feel worthless and unimportant.				

**42. Trust in fate-that things have a way of working out for the best.**				
**43. Use my past experience to try deal with the situation.**				
**44. Try to forget the whole thing.**				
**45. Just take nothing personally.**				
**46. Become irritable or angry.**				
**47. Just give the situation my full attention.**				
**48. Just take one step at a time.**				
**49. Criticise or blame myself.**				
**50. Simply and quickly disregard all irrelevant information.**				
**51. Pray that things will just change.**				
**52. Think or talk about the problem as if it did not belong to me.**				
**53. Talk about it as little as possible.**				
**54. Prepare myself for the worst possible outcome.**				
**55. Feel completely calm in the face of any adversity.**				
**56. Look for sympathy and understanding from people.**				
**57. See the thing as a challenge that must be met.**				
**58. Be realistic in my approach to the situation.**				
**59. Try to think about or do something else.**				
**60. Do something that will make me feel better.**				
